# External iliac vein compression and lower-extremity swelling caused by an iliopectineal ganglion: a case report

**DOI:** 10.1186/s13256-019-2223-4

**Published:** 2019-09-16

**Authors:** Kiyokazu Fukui, Ayumi Kaneuji, Norio Kawahara

**Affiliations:** Department of Orthopedic Surgery, Kanazawa Medical University, 1-1 Daigaku, Uchinada-machi, Kahoku-gun, Ishikawa, 920-0293 Japan

**Keywords:** Iliopectineal ganglion, External iliac/femoral vein, Leg swelling

## Abstract

**Background:**

A hip joint ganglion is a rare cause of lower-extremity swelling.

**Case presentation:**

We report a case of a Japanese patient with ganglion of the hip with compression of the external iliac/femoral vein that produced signs and symptoms mimicking those of deep vein thrombosis.

**Conclusions:**

Needle aspiration of the ganglion was performed, and swelling of the lower extremity promptly decreased. At 7.5 years after aspiration, there was no recurrence of swelling of the leg. Although the recurrence rate for ganglions after needle aspiration is high, it is worthwhile trying aspiration first.

## Introduction

Ganglion cysts of the hip joint are rare. Compression of the femoral vein by a synovial or ganglion cyst of the hip joint results in leg swelling resembling that caused by deep vein thrombosis [1]. We present a case of a patient with lower-limb swelling due to compression of the external iliac/femoral vein by a hip joint ganglion.

## Case presentation

**Fig. 1:**
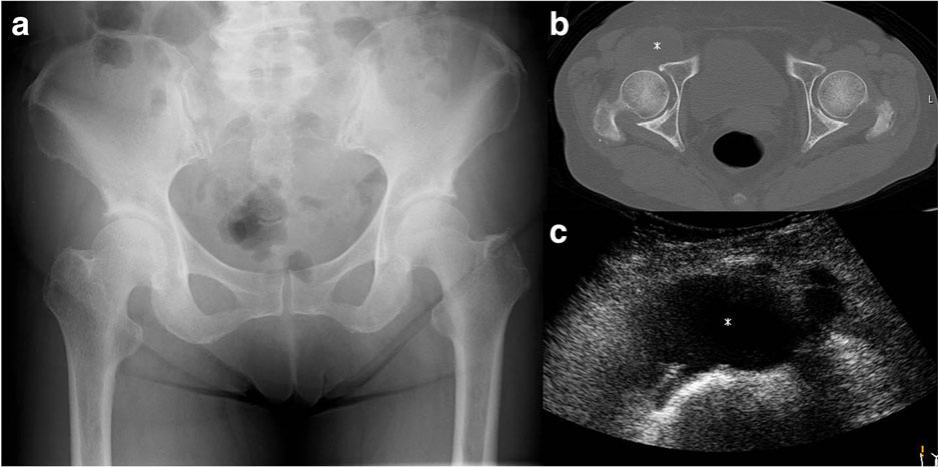
**a** An initial anteroposterior radiograph showed no significant findings. Computed tomography (**b**) and ultrasonography (**c**) showed a cystic mass (asterisk) arising from the right hip joint and compressing the femoral vein

**Fig. 2:**
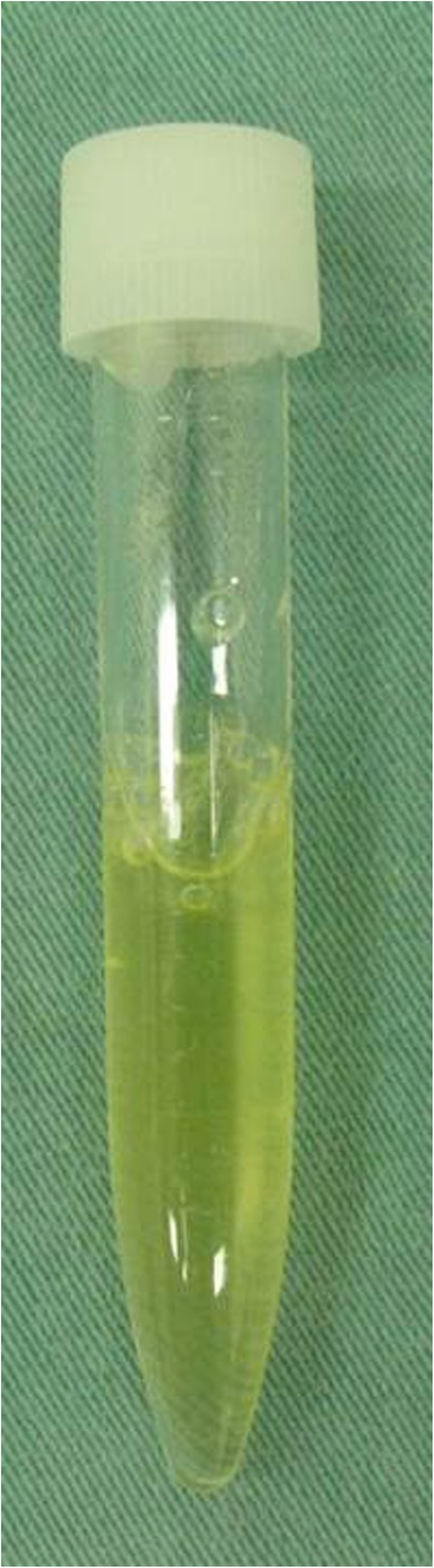
Macroscopic appearance of aspirated fluid from the cyst

**Fig. 3:**
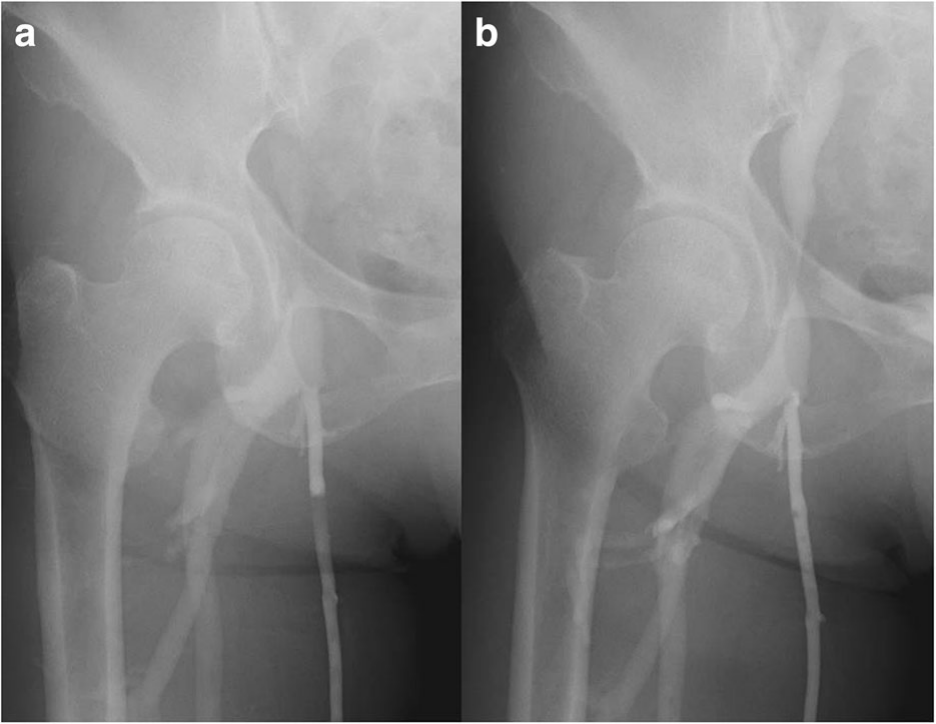
**a** Ascending venography demonstrated external compression and blockage of the right external iliac vein. **b** The blockage resolved immediately after needle aspiration

**Fig. 4:**
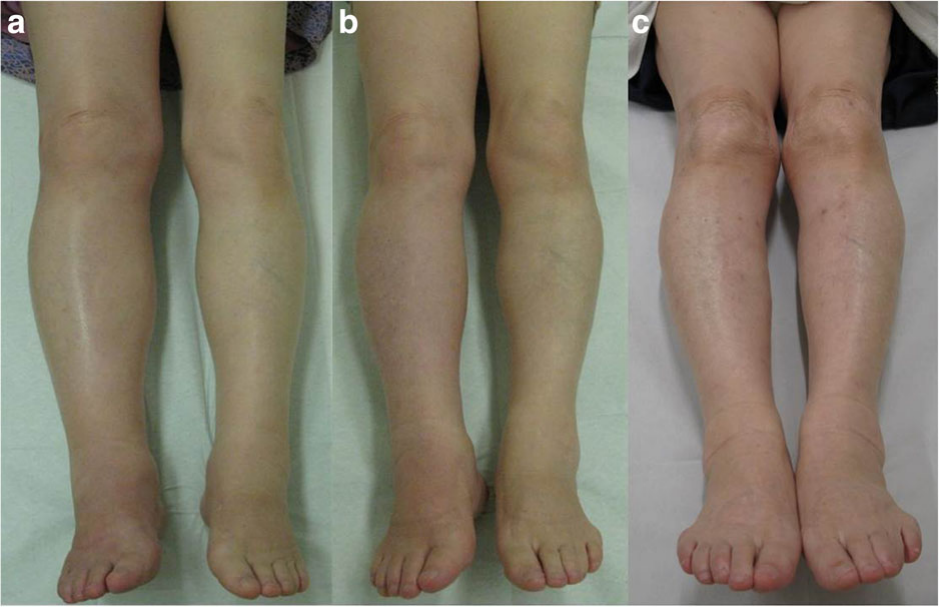
At the first visit, the patient’s right lower extremity was swollen and had a larger circumference than that of the left lower extremity (**a**). The lower extremities at 10 days after needle aspiration (**b**) and the leg silhouettes at 7.5 years afterward (**c**) were symmetrical

**Fig. 5:**
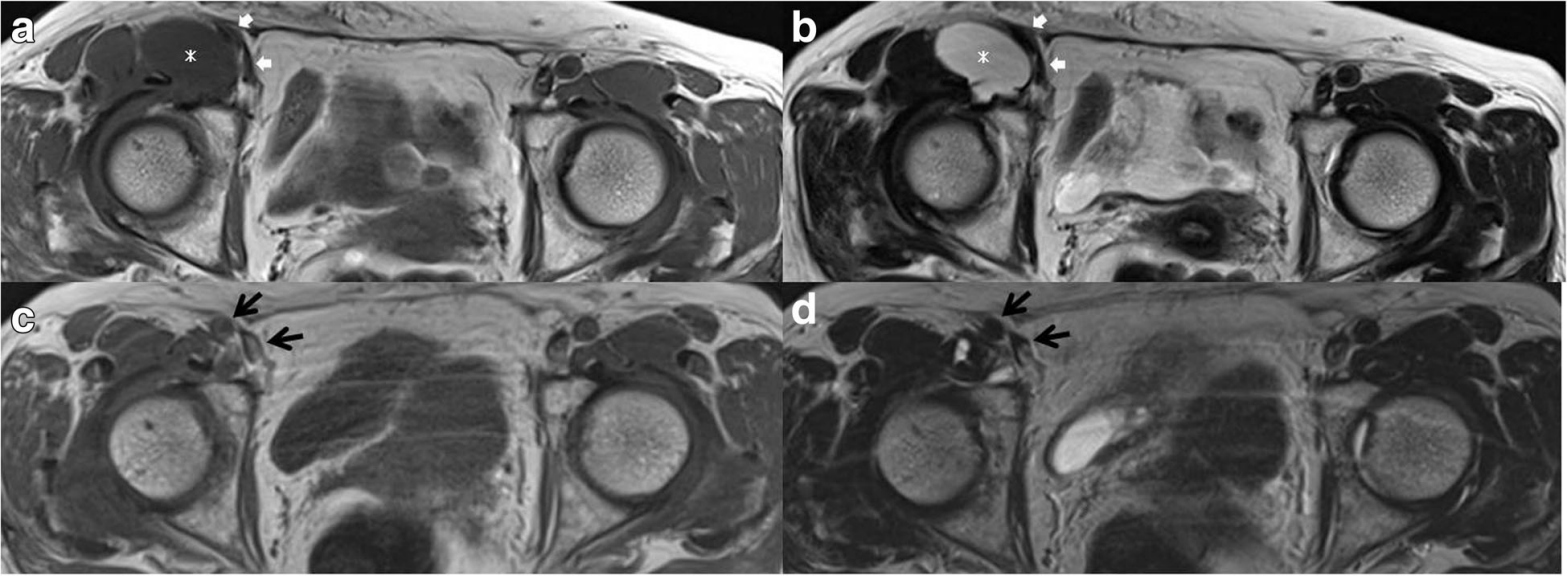
Axial T1-weighted (**a**) and T2-weighted (**b**) magnetic resonance imaging performed before needle aspiration showed that the cystic mass (asterisk) had increased in size to 45 × 25 mm. The cystic mass in the right groin had compressed femoral vessels (white arrows). Axial T1-weighted (**c**) and T2-weighted (**d**) magnetic resonance images obtained at 7.5 years after needle aspiration revealed that the cyst had considerably shrunk in size and femoral vessels had been released from the compression (black arrows)

An 80-year-old Japanese woman came to our department because of gradual-onset swelling of the entire right lower extremity; it had been swollen for 3 months. She reported mild dullness in the right leg. Ultrasonography did not show venous thrombi, but it did show a cystic lesion measuring 3 to 4 cm in diameter posterior to the right femoral vein. Although no significant findings were seen on an initial anteroposterior computed tomographic scan and magnetic resonance imaging of the right hip revealed, in both low T1-weighted and high T2-weighted signal intensity, a cystic lesion arising from the anteromedial aspect of the acetabulum (Fig. 1a, b). We performed needle aspiration of the cyst with a 21-gauge needle under the guidance of an x-ray image intensifier while also performing ascending venography. Aspiration yielded approximately 6 ml of clear, jelly-like fluid (Fig. 2). Venography revealed external compression and narrowing of the right external iliac/femoral vein. Venous flow proximal to the portion of the compression was restored immediately after aspiration (Fig. 3), and the swelling in the right lower extremity promptly decreased (Fig. 4a, b). Therefore, we did not need to extirpate the ganglion cyst. By 7.5 years after aspiration, swelling had not recurred in the lower extremity (Fig. 4c), though magnetic resonance imaging showed that the cyst had remained at a small size (Fig. 5c, d). To our knowledge, this follow-up period is the longest reported so far after simple needle aspiration.

## Discussion and conclusions

Ganglion cysts can develop on any joint lining or tendon sheath, although they usually occur at the wrist, ankle, or knee. Ganglion cysts of the hip joint are not common and are usually accompanied by hip disorders such as rheumatoid arthritis or osteoarthritis, or by trauma. Cysts in the hip are benign synovial protuberances resulting from high pressure in the joint. Their usual clinical presentation involves an inguinal mass and groin or thigh pain. However, it is unusual for such cysts to cause compressive symptoms. Compression of the femoral or iliac vein causes leg swelling resembling that caused by deep vein thrombosis or lymphatic edema [1–6]. Gale *et al.* were the first to report that femoral ganglia cause deep vein obstruction and leg swelling [7]. Colasanti *et al*. reported on a case of their own and summarized findings of 27 previously reported cases of lower-limb swelling caused by synovial cysts of the hip joint. The mean age of these patients was 62 years (range, 35–80 years), and 60% of the patients were women. In 55% of the cases, the joint cysts were accompanied by either rheumatoid arthritis or osteoarthritis. Surgery was the treatment of choice in 70% of the cases, and in the remaining cases, the patients were treated with needle aspiration. Lower-limb swelling recurred in 37% of the patients initially treated with needle aspiration, whereas there was recurrence in only one patient treated surgically [8].

Treatment of ganglion cysts depends on their size, location, and symptoms, and options include observation, needle aspiration, and surgical extirpation. Asymptomatic cysts are often treated with observation. Once a cyst in the hip joint causes compressive symptoms, the ideal treatment is surgical excision because the recurrence rate for cysts treated with needle aspiration is high. However, needle aspiration is easier to perform and is less invasive than surgery [4]. In our patient, we treated the mass with needle aspiration only; therefore, we did not perform a histologic examination. The mass contained a yellowish, transparent, jelly-like fluid that is typical of ganglion cysts, so that confirmed our diagnosis. After the patient’s leg swelling promptly decreased, she did not want to undergo surgery. Surgery has been recommended for the treatment of synovial cysts because such cysts frequently recur after needle aspiration. However, previous reports have shown that in some cases, simple needle aspiration resolved problems caused by synovial cysts [9, 10]. Because our patient had no symptoms at 7.5 years after needle aspiration despite residual cyst, we suggest that needle aspiration should be tried first.

## Data Availability

The datasets used and/or analyzed during the current study are available from the corresponding author on reasonable request.
